# Dr. Directus DeForest Cole

**Published:** 1915-07

**Authors:** 


**Dr. Directus DeForest Cole,** Hahnemann of Chicago 1881, died at his home in Newark, N. Y., May 24, aged 60.

